# Fresh Food Rx: Evaluating the Impact of a Produce Prescription Program on Engagement and Well-being Using the RE-AIM Framework

**DOI:** 10.1007/s11606-025-09687-0

**Published:** 2025-07-14

**Authors:** Rachel Zimmer, Ashley Strahley, Diane Shenberger, Deepak Palakshappa, Lindsey Abdelfattah, Sarah A. Birken, Roger Vilardaga, Charlotte Crotts, Amresh Hanchate

**Affiliations:** 1 Department of Implementation Science, Division of Public Health Sciences, Wake Forest School of Medicine, 529 Vine Street, 4th Floor, Winston Salem, NC 27101, USA;; 2 Department of Social Sciences and Health Policy, Division of Public Health Sciences, Wake Forest University School of Medicine, 529 Vine Street, 4th floor, Winston Salem, NC 27101, USA;; 3 YMCA of Northwest NC, Winston Lake Family YMCA/REACH Center, Winston Salem, 901 Waterworks Rd, NC 27101, USA;; 4 Department of Internal Medicine, Wake Forest School of Medicine, Downtown Health Plaza, 1200 N Martin Luther King Jr Dr, Winston-Salem, NC 27101, USA;; 5 Department of Epidemiology and Prevention, Division of Public Health Sciences, Wake Forest University School of Medicine, Downtown Health Plaza, 1200 N Martin Luther King Jr Dr, Winston-Salem, NC 27101, USA;; 6 Section on Gerontology and Geriatric Medicine, Department of Internal Medicine, Wake Forest School of Medicine, Winston Salem, USA, Ground Floor, Administration Suite, NC 27157

**Keywords:** food is medicine, food insecurity, produce prescription, Implementation science, Medicaid, behavior change theory, North Carolina, qualitative research, RE-AIM

## Abstract

**INTRODUCTION:**

Food insecurity (FI) affects 13.8% of the USA, disproportionately afflicts low-income and minority populations, and is associated with increased risks of chronic health conditions. Produce prescription (PRx) programs, a Food Is Medicine intervention, have emerged as a promising way to address FI and improve the health of participants with limited access to healthy foods and chronic metabolic conditions, yet PRx implementation in practice remains poorly understood.

**METHODS:**

We used the Reach, Effectiveness, Adoption, Implementation, Maintenance framework to inform semi-structured interviews with 15 participants enrolled in a 12-month PRx intervention that provided weekly produce deliveries, personalized nutrition coaching, and community-based education sessions to alleviate food insecurity and support dietary behavior change. Interviews explored the intervention’s implementation and impacts. We analyzed interview data using a reflexive thematic analysis approach.

**SETTING:**

Academic health system in North Carolina.

**RESULTS:**

Participants expressed a variety of motivations for participation (reach). Improved dietary knowledge, community engagement, improvements in health, and motivations to prioritize self-care were viewed as benefits of participation (effectiveness). Engagement was facilitated by social connection as well as the financial benefit and consistency of receiving weekly produce boxes. Barriers to full engagement in PRx (implementation) included logistical, transportation, and financial constraints. Participants expressed a strong commitment to maintaining healthy eating habits, but many were unsure how they would do so because of systemic barriers to healthy food (maintenance).

**CONCLUSIONS:**

Our study generated novel evidence regarding PRx implementation. We found that PRx implementation was limited by logistical and financial constraints and facilitated by consistent produce access, relationships with the staff and fellow peers, and community-based education. Our findings suggest that flexible produce delivery options, individual-level dietary counseling, and community-located educational sessions enhanced PRx implementation. Future studies should examine strategies to support engagement and sustained behavior change and evaluate mechanisms for stable program implementation.

## INTRODUCTION

Food insecurity (FI), defined as limited or uncertain access to adequate nutrition, is a significant public health challenge that has intensified in recent years, with prevalence increasing from 10.2% to 13.8%, between 2021 and 2023 [1, 2]. This issue disproportionately affects households with children, low income, and minority populations, exacerbating chronic health conditions and contributing to disparities in healthcare utilization and to over 500,000 preventable deaths annually [3–7].

The health impacts of FI are multidimensional, increasing the risk of cardiometabolic disorders, depression, and decreased engagement in healthy behaviors [3, 7–12, 14]. Consequently, interventions designed to address FI can yield improvements in both physical and mental health outcomes, particularly among individuals facing structural and systemic barriers that limit their access to nutritious foods [10–13].

In response, national attention has increasingly focused on Food Is Medicine (FIM) approaches, food-based interventions integrated into healthcare settings targeting patients with specific health conditions and social needs [14, 15]. These programs have been exemplified by the White House Conference on Hunger, Nutrition, and Health in 2022 and funded by the Nutrition Incentive Hub’s Gus Schumacher Nutrition Incentive Program (GusNIP) [14–16]. Within the FIM movement, produce prescription programs (PRx), which provide subsidized or free access to fresh fruits and vegetables accompanied by nutrition education and behavioral support, have emerged as a promising intervention [16–26]. Previous studies indicate high participant satisfaction and demonstrate feasibility and acceptability of PRx programs, noting improved access to healthy foods, dietary knowledge, health outcomes, and motivation among participants [19, 22–26]. For example, Fischer et al. found home-delivered produce prescriptions feasible [22] and Schlosser et al. emphasized the significance of interpersonal relationships in facilitating successful implementation [23].

However, existing research on PRx programs lacks a comprehensive understanding of participant-centered barriers and facilitators to adoption and sustained engagement [21–25]. Specifically, there are notable gaps concerning how to effectively identify and engage target populations, how nutrition education and personalized coaching contribute to outcomes, and which implementation strategies most effectively address participant-facing challenges, such as logistical or financial constraints [14, 15, 22–26]. Addressing these gaps can help to improve PRx program effectiveness, sustainability, and scalability.

Our study directly addresses these implementation knowledge gaps by systematically examining participant experiences and identifying specific implementation barriers and facilitators using the Reach, Effectiveness, Adoption, Implementation, Maintenance (RE-AIM) framework [27–29]. By analyzing the Fresh Food Rx PRx program, a 12-month intervention involving weekly produce box deliveries, personalized nutrition coaching, and community-centered educational events, our objectives were to (1) understand motivations for participation and strategies for reaching target populations, (2) examine how specific program components contribute to participant engagement and health outcomes, (3) identify patient-facing implementation facilitators and barriers, and (4) investigate factors influencing sustained healthy eating behaviors after program completion. By examining participant experiences across these domains, we aimed to generate practical insights for healthcare systems implementing similar programs and advance understanding of how to effectively deliver and sustain PRx interventions in clinical settings [21–25]. This evidence is particularly timely given increasing national interest in FIM approaches [14, 15] and need for evidence-based implementation guidance as programs scale.

## METHODS

### A Produce Prescription Program Intervention in North Carolina

The Fresh Food Rx (FFRx) program was a 12-month produce prescription (PRx) intervention that enrolled 112 participants and compared outcomes to a matched cohort of 92 participants receiving Supplemental Nutrition Assistance Program (SNAP). Fresh Food Rx was launched in collaboration with Atrium Health Wake Forest Baptist, the YMCA of Northwest North Carolina, and The Produce Box (Raleigh, NC). Participants were eligible for the program if they experienced food insecurity, had Medicaid insurance and a metabolic condition, and resided in Forsyth County. They were enrolled through AHWFB clinical team referrals or self-referrals, including flyers posted at YMCA locations. This intervention was designed to alleviate food insecurity among individuals in Forsyth County, NC, where the prevalence of FI is notably higher (14.5%) than the national average 13.8% [30, 31] (see Table 1).

This intervention was developed using behavioral science frameworks —the Behavior Change Wheel (BCW) and Theoretical Domains Framework — to ensure a comprehensive participant-centered approach addressing both individual and structural barriers to healthy eating [19, 20, 32, 33] (see Table 2). These frameworks guided the design to systematically target core behavioral components: capability, opportunity, and motivation (COM-B model), and psychological determinants such as knowledge, social norms, and self-efficacy [32, 33]. Prior to this study, a pilot implementation (06/2020–10/2021) informed adjustments to enhance program effectiveness, scalability, and acceptability [19, 20].

Participants received weekly deliveries of produce boxes of regionally sourced fresh fruits and vegetables. Personalized nutrition coaching sessions were offered using motivational interviewing techniques, including an initial in-person baseline visit, followed by virtual one-on-one sessions primarily during the first 3 months of the intervention, with occasional check-ins throughout the remaining program duration. Additionally, participants had access to both community-based and virtual educational events designed to engage participants in knowledge and skill-building activities to improve healthy dietary behaviors. These intervention components collectively aimed to mitigate practical barriers (e.g., transportation, financial constraints), cognitive barriers (e.g., dietary knowledge gaps), and emotional barriers (e.g., motivation and self-efficacy) associated with food insecurity [34–40] (see Table 2).

### Study Design

We employed a qualitative descriptive design as part of a larger mixed methods evaluation to deeply explore participants’ lived experiences and perspectives of the Fresh Food Rx program [41]. This method was selected to preserve the richness of participant narratives without overinterpretation, and to understand how specific intervention components influenced their dietary behaviors, overall well-being, and participant engagement [41, 42].

### Sampling and Recruitment

We used purposive sampling to recruit a diverse subset of 15 English-speaking participants enrolled in Fresh Food Rx, intentionally including a demographic mix representative of age, gender, and racial diversity. Emphasis was placed on including individuals aged 60 or older to capture the unique perspectives of older adults. Participants who had previously provided permission to be contacted for interviews to gain their perspectives on the Fresh Food Rx program were contacted via phone by a study team member, unknown to the participant to reduce bias. Interviews were conducted as semi-structured, 1:1 telephone interviews to promote accessibility for participants.

### Data Collection

We designed the semi-structured interviews based on the RE-AIM framework to systematically assess the intervention’s reach, effectiveness, adoption, implementation, and maintenance [27, 42]. Interviews explored motivations for participation, barriers and facilitators to healthy eating, perceptions of health impacts, and suggestions for program improvement—with flexibility to capture participants’ lived experiences (see Table 3).

By exploring these factors, we hoped to gain insight into what drives sustained participation, helping uncover the components of the program that participants found most valuable.

Telephone interviews were performed one-to-one with participants by a trained and experienced qualitative researcher who has published several qualitative studies (AS), lasted between 10 and 39 min, were audio recorded, and transcribed verbatim. Data collection continued until thematic saturation was reached [41]. Field notes were made after the interviews to capture researcher perspectives and insights. The Institutional Review Board of Wake Forest University School of Medicine approved this study (IRB00089384).

### Data Analysis

Data were analyzed using reflexive thematic analysis, allowing us to systematically explore participants’ perceptions and experiences while remaining open to emergent themes [44]. Interview transcripts were compared to the audio and edited for accuracy by the study team. Two qualitative researchers (AES and RPZ) independently reviewed and coded the transcripts, beginning with open coding and then deductive coding informed by the RE-AIM framework, and adding inductive codes as new themes surfaced during analysis [27]. This combination of deductive and inductive coding permitted both a structured and flexible exploration of the data, enriching our understanding of the participant experiences and engagement with the FFRx program [41].

The coded data were managed with ATLAS.ti version 23 software, and the researchers met weekly to review, discuss, and resolve discrepancies, iteratively refining the codebook to reflect the emerging patterns [43]. Summaries were then synthesized using reflexive thematic analysis and mapped onto the RE-AIM framework, preserving the participants’ lived experiences [27, 41].

## RESULTS

Semi-structured interviews were conducted with 15 study participants who received the FFRx intervention. Half were over the age of 60 (50%), 67% were female, and 47% were Black (see Table 4). All participants, except one person, participated in at least two nutrition coaching sessions, and ten participants engaged in at least one educational event offered by the program.

### Qualitative Themes

We identified five main primary themes: (1) participants expressed a variety of motivations for participation (reach); (2) improved dietary knowledge, community engagement, observed improvements in health, and motivations to prioritize self-care were key benefits of program participation (effectiveness); (3) several factors influenced program engagement including personal connections, financial benefits, and sustained access to weekly produce (adoption); (4) while participants recognized the program’s benefits, several barriers hindered full participation (implementation); and (5) participants expressed a strong commitment to maintaining healthy eating habits after the program’s discontinuation, but many were unsure how they would do so because of systemic barriers to healthy foods (maintenance). Each theme provides insight into the nuanced ways the program influenced participants’ dietary choices, health perceptions, and community connections (see Table 5).

#### Participants Expressed a Variety of Motivations for Participation (Reach)

Participants’ initial reasons for joining Fresh Food Rx included desired financial relief, access to fresh foods, an aspiration to improve health outcomes, or encouragement to participate from their healthcare providers. Many were referred by healthcare teams or informed through social media, flyers, or word of mouth. The program’s ability to provide consistent access to weekly produce and reduce financial strain was particularly impactful, as participants reported that they could allocate their limited resources to other essentials. One participant noted, “Getting the boxes…I was able to buy other things I needed since I didn’t have to buy eggs, bananas, and apples.” (P8). For others, the opportunity to learn about nutrition and adopt healthier eating habits to manage specific health conditions, such as diabetes or hypertension, was key. A participant shared, “[I wanted] to get the weight off so I can eat more healthier… find other ways to eat that’s better so I can get the weight off.” (P03).

Participants advised that the team should consider diverse recruitment strategies including word of mouth, visits to low-income apartment complexes, social media live events, news channels, direct mail, and flyer distribution at popular shopping locations (e.g., Goodwill, grocery stores) in the future. One participant advised, “The best way to tell’em is the way I got the information—through the doctor’s office. Those are the people that need it most.” (P16).

#### Improved Dietary Knowledge, Community Engagement, Observed Improvements in health, and Motivations to prioritize Self-Care Were Key Benefits of Program Participation (Effectiveness)

##### Participants gained improved dietary knowledge and skills during program participation.

Participants described a variety of benefits from the program stemming from their engagement in the FFRx program, including increased nutritional knowledge and skills, improved health, and meaningful social connections. Many participants reported significantly increasing their fruit and vegetable consumption, often replacing processed foods with fresh produce as staples in their diets. One participant noted, “When I get my food stamps, instead of going out and buying junk… I’d rather buy lots of fruits and vegetables.” (P12). For some, their produce intake doubled compared to before the program.

The knowledge gained empowered some to seek out information about their dietary needs independently. One participant shared the impact coaching sessions, “She gave me ideas to go on the search engine myself and look things up. It was very helpful.” (P01). A participant shared, “I remember them showing different foods and how eating those nutrients help you with lowering your A1C.” (P11). Many participants also reported adjusting their portion sizes and eating more regularly, contributing to more consistent meal patterns. Even those who did not drastically change their dietary patterns found the weekly produce box helpful, serving as consistent reminders to prioritize fresh, healthy ingredients.

Participants valued the opportunity to explore new foods and learn healthier cooking techniques. Educational events and coaching sessions inspired creativity in the kitchen, with participants trying recipes that incorporated produce provided by the program, such as zucchini and spaghetti squash, in balanced meals. Several described transitioning to healthier cooking methods, like baking or air frying and using seasoning for flavor. One participant shared, “My daughter, she lives with me, and she was encouraged to try new things for us to eat instead of having the same old thing…was able to try new things and actually found that we love some stuff.” (P11). For some, cooking became a form of self-care or a way to bond with family, adding enjoyment to meal preparation and dining.

The variety of produce in the program allowed participants to explore and enjoy foods they had never tried before. For many, the opportunity to experiment with new ingredients brought an unexpected sense of adventure and satisfaction. One participant stated, “I never in my life seen a cucumber. Never. I decided to make a salad out of it, and then make a pasta out of it after I ate the salad…it turned out to be pretty good.” (P12). Others appreciated trying fresh eggs for the first time or learning how to incorporate new items like pears into meals, enhancing their culinary skills and enjoyment of food.

##### Participants were motivated to prioritize self-care and expressed improvements in physical and mental health.

Many participants reported feeling less sluggish, more energetic, and overall healthier as they adopted and improved eating habits. One participant shared, “It helped me to break from bad eating habits…I lost weight. When I went into the program, I was like 247 lb. I’m down to 218 now.” (P18). Others experienced specific health benefits, such as reduced joint pain and increased energy levels, which motivated them to continue with the program. Another participant, who had recently lost her husband, described how the program helped her regain motivation, saying, “I has gotten me to a point that I feel more motivated now. Before, I was just lying in bed, and I wanted just to do nothing, just shut myself off completely from the world.” She further emphasized the transformative nature of the program, saying, “I think the program works wonders. You change your life. It changes your life.” (P12).

##### Participants experienced an improved sense of community and social connections.

Group educational events and coaching sessions created a supportive environment where participants shared experiences, learned from one another, and felt less isolated. This community aspect contributed to participants’ motivation to continue prioritizing their health. One participant recalled, “I loved it. I always looked forward to it because it was someone to talk to other than a therapist… That was helpful.” (P03). Participants felt supported by the coaching staff and emphasized the program’s supportive environment, “They were so nice, so friendly. [I enjoyed] the educational stuff, I loved the program.” (P18).

##### Program benefits extended to others.

The benefits of the program often extended beyond individual participants to their households and communities. Several participants noted that others in their households began adopting healthier eating habits because of the program. For example, a mother shared, “It not only helped me, but it helped me make sure that my kids were participating with me… We all started eating healthy.” (P12). A grandmother humorously recounted how her grandchildren would often eat the produce box contents before she could, including blueberries she had intended for pancakes. She enjoyed teaching her granddaughter to cook with new ingredients, like making egg benedict and omelets. (P08) Additionally, some participants shared excess produce with neighbors, who also had barriers to access to healthy foods. One participant remarked, “A lot of people [around here] don’t have access to this and it’s good.” (P15).

#### Several Factors Influenced Program Engagement Including Personal Connections, Financial Benefits, and Sustained Access to Weekly Produce (Adoption)

Personal connections formed through nutritional coaching and educational events played a key role in keeping participants engaged. Many participants appreciated the attentiveness and support from both dieticians and staff, describing the sessions as empowering and educational; for some, the program sessions became a source of motivation and companionship. One participant expressed, “I loved it. I always looked forward to it…someone else to talk to other than just having a therapist. Even if we did talk about other things, she listened.” (P03). While some participants preferred in-person coaching for the face-to-face interaction, others found virtual sessions more convenient, highlighting the program’s flexibility. This sense of community extended to educational events, where participants enjoyed learning together, reducing feelings of isolation and strengthening social bonds. One participant noted, “Meeting person to person, I feel more connected.” (P01).

Participants recommended that the team introduce additional in-person group activities and peer mentoring opportunities to foster continued connections among participants. One participant suggested pairing new participants with those who have had success in the program, “Maybe pairing people who’ve had success, letting those people come together and invite people who may not be aware of the program… I don’t know if you all have had this before, but having some of the people who contribute to connect with some of the participants to, maybe, teach them gardening skills” (P04).

##### The weekly delivery of fresh produce boxes provided stability and incentive to maintain healthy eating habits.

Participants looked forward to the produce box deliveries, which served as a tangible reminder to focus on their health. The quality and locally sourced nature of the produce added to its appeal, with one participant describing it as, “just the blessing of receiving fresh, locally grown, organic food on a weekly basis.” (P04). Another participant explained that receiving the box was a stabilizing factor that kept him “focused” on healthy eating.

##### The program’s financial benefits alleviated economic burden.

Several participants reported that receiving the produce boxes eased their grocery expenses, allowing them to purchase a wider variety of produce and other essential items. One participant explained, “[I was] able to provide for myself with more things, as far as my medication and personal items, things I wasn’t able to get [before].” (P03). Some participants also explained how the boxes helped alleviate the mental strain of managing limited funds, as participants knew they could rely on the weekly deliveries for staples. Another participant stated, “The mental part of it is if you got enough in that box, you don’t have to worry about getting it with your EBT card.” (P16).

#### While Participants Recognized the Program’s Benefits, Several Barriers Hindered Full Participation (Implementation)

Key challenges for many participants included scheduling conflicts, transportation difficulties, dietary restrictions, and financial constraints. These issues, particularly among those with chronic health conditions, highlighted a need for increased flexibility and support from our study team.

##### Scheduling Conflicts and Accessibility.

Several participants faced scheduling conflicts that prevented attendance at educational events or coaching sessions, often due to medical appointments. One participant reflected, “They sent the flyers out, but all the times were right around the time a of doctor appointment. I got the cancer doctor, urologist, and the primary doctor.” (P16). Flexible scheduling or alternate engagement methods, such as virtual or asynchronous options, were suggested by some to help address these conflicts. Participants also recommended that the program provide enhanced educational offerings, including classes on healthy meal preparation, group exercise, gardening workshops, or group coaching sessions. One participant suggested, “You could always do it [teaching] on a Zoom, and then give us the information to tune in.” (P05). Participants also suggested the team use tools such as flyers to help inform participants about educational events and program updates. Even though the team used flyers, some participants did not receive them consistently, “Flyers were not always in boxes…so may not have realized an event was happening.” (P04). They suggested implementation of reminder calls or messages for upcoming events and sessions to increase attendance and engagement.

##### Transportation and Financial Barriers.

Transportation was a barrier that impacted the full engagement of participants, affecting their ability to attend in-person sessions. Some expressed a preference for phone or virtual coaching. However, despite this complication, other participants preferred in-person sessions because of the personal connections they made. Their experiences reflect several studies that suggest FIM programs not only improved physical health, but also encouraged social connectedness [25, 26, 44]. Many expressed that it is important for this program to provide transportation to and from events and offer virtual options for coaching and educational sessions to accommodate participants with mobility or scheduling challenges. “If they could come and get me and bring me back home, that’d be okay. I gotta worry about riding the bus and have to walk back.” (P06). Participant also advised that the team offer educational events at various times to accommodate different schedules.

##### Dietary Limitations and Produce Accessibility.

Some participants faced dietary restrictions, preventing them from fully utilizing the produce boxes due to specific health-related needs. For instance, participants with sensitivities to acidic foods or difficulties with certain textures faced limitations in using all the items provided. Participants recommended that the program offer more variety and customization options in produce boxes to cater to individual preferences and dietary restrictions, and consider increasing the quantity of produce, especially for larger households. One participant suggested, “I think they should offer the person to be able to pick what items they want.” (P08). The produce boxes had a retail value of $25, which felt insufficient for some participants, as grocery prices increased by 5% on average and SNAP benefits decreased to pre-pandemic levels during the program time [47]. Another participant provided insight on their experiences with the produce box contents, “They could provide more produce than what they have been giving me. […] It didn’t last very long. By the time I made a couple of dishes out of ‘em, they were gone, and I wish there was more produce in them so they would last a whole lot longer.” (P09).

#### Participants Expressed a Strong Commitment to Maintaining Healthy Eating Habits After the Program’s Discontinuation, But Many Were Unsure How They Would Do So Due to Systemic Barriers to Healthy Foods (Maintenance)

Many participants received SNAP benefits, and some highlighted their reliance on SNAP benefits to afford nutritious food. One participant explained concerns about the affordability of healthy foods, “Food stamps …That’s the only way, every month. I’m out of eggs and vegetables, ‘cause I ate ‘em all. I’m waiting for tomorrow.” (P06). Others shared similar concerns, with one participant estimating they were “30% likely” to maintain eating healthy due to financial constraints. (P11). Many noted that while food stamps helped some, the benefits were not sufficient to meet all household needs, especially for families with children. They advised that the program team should consider participants’ financial constraints by exploring ways to make a produce prescription program affordable. “Keep it going …find a way to fund it for people who don’t have the finances; it helps me tremendously.” (P08).

Transportation barriers further complicated participants’ ability to shop for healthy produce regularly. One participant expressed a desire to visit farmers’ markets but cited the lack of transportation as a significant, stating, “I’d really love to get to the farmer’s market, but from where I live and since I don’t have the transportation, that’s a problem.” (P13). Others relied on grocery delivery services or struggled using public transportation to carry groceries, which was often cumbersome and time-consuming.

Despite uncertainties about maintaining healthy eating habits post-program, many expressed a strong commitment to continue. One participant stated, “I’ll be eating just the way I am now…eating fruits and vegetables like I do now. I’m used to it, so I have to do it.” (P19). Another participant attributed the boxes to helping them stay focused and worried that losing the support might lead to a decline in dietary habits: “Without the box, my focus would slip.” (P16).

Participants discussed various strategies to sustain their healthy diets. Some planned to grow their own produce or shop strategically, focusing on sales, bulk buying, or exploring grocery delivery services. Gardening, including hydroponic methods, emerged as an affordable alternative to supplement diets. Others explained that food stamps were crucial to supplementing their diets. One participant explained, “I wasn’t able to afford things, so if I could grow my vegetables, I could afford to buy some fruit…Whatever I couldn’t grow, I would have to buy.” (P11). Participants were hopeful that the program would continue and asked for the team to consider ways to provide continued support with follow-up sessions to help participants maintain healthy eating habits.

## DISCUSSION

Our analysis of a PRx implementation using the RE-AIM framework provided insights into participant experiences and highlighted several important considerations for future practice [27]. First, our findings indicate that healthcare provider referrals combined with multiple outreach channels were effective strategies for reaching target populations, leveraging trust inherent in patient-provider relationships. Second, participant engagement and adoption of healthier behaviors appeared to be supported by flexible delivery options, personalized nutrition counseling, community-based education, and integrated social support. Third, the intervention faced several barriers, including participant transportation challenges, scheduling conflicts, communication gaps, and limited customization of produce boxes, which impacted full participation. Finally, participants reported significant sustainability concerns related to maintaining healthy eating habits due to the lack of affordable produce post-intervention and uncertainties regarding continued funding.

### Contribution to Understanding of PRx Implementation Strategies

The study contributes to existing literature by emphasizing the benefits of theory-informed program design through frameworks like the Behavior Change Wheel (BCW) and Theoretical Domains Framework (TDF) [34, 35]. Such structured approaches enable the identification and mitigation of barriers beyond logistical and community-level factors previously reported. Prior research has highlighted the value of home delivery [36] and interpersonal relationships in facilitating program engagement. [25, 26] Our identification of barriers extends Newman and Lee’s findings on PRx implementation challenges in Georgia, providing examples of how flexibility in delivery methods and structured community-building activities can address practical barriers like transportation and scheduling [35].

Our study also underscores the importance of maintaining flexibility and responsiveness to participant feedback throughout the program implementation. The integration of social connection opportunities, alongside practical support mechanisms, appears particularly beneficial in overcoming traditional barriers to program participation [25, 26, 36–40]. These findings align with evidence advocating multilevel, participant-centered strategies, especially critical for populations experiencing multifaceted social and structural barriers [36–38, 40, 45, 49].

### Policy Implications and Sustainability

Integrating PRx programs into broader healthcare models requires considerable infrastructure. Key components include standardized clinical assessment and referral pathways integrated into routine clinical workflows, [18] strong community partnerships, [18, 35] closed-loop referral systems, and robust evaluation frameworks [18, 35, 46]. Additionally, community-level infrastructure development necessitates diverse expertise of partners, adequate technological resources, and operational capacity, including transportation solutions and storage facilities for produce storage. This study was small in scale and findings are preliminary. Further evidence from larger-scale evaluations would help identify infrastructural strategies that could be impactful to scale programs effectively.

Medicaid 1115 waivers represent a potential funding mechanism, allowing healthcare systems to incorporate nutritional services for high-need populations [46]. Examples, such as North Carolina’s Healthy Opportunities Pilot, illustrate promising pathways for leveraging Medicaid funds to support interventions targeting social determinants of health [46, 58]. However, the sustainability and scalability of such programs are uncertain and require further exploration as well as rigorous evaluation [46, 58].

In addition to Medicaid-funded initiatives, North Carolina has implemented several state- and county-level produce prescription programs that offer alternative models for PRx implementation. For instance, the Eat Well program, managed by Reinvestment Partners, provides participants with monthly stipends to purchase fruits and vegetables at participating retailers statewide [59]. This program has demonstrated success in increased produce consumption among low-income populations. Other community-based programs, such as the Farm Fresh Produce Prescription by the Appalachian Sustainable Agriculture Project, have tailored their approaches to meet the specific needs of local communities [60]. These programs highlight the potential for state and county-funded interventions to complement larger healthcare models in promoting healthy eating habits.

### Limitations

While the Fresh Food Rx program provided significant support through nutrition coaching, education, and produce delivery, it is important to acknowledge that these components alone may be insufficient to fully sustain behavior change once the program ends. Our findings should be interpreted considering several limitations. First, participants were able to opt into the interviews, which may have introduced bias toward those with more favorable views or greater engagement in the program. Second, the study was conducted within a single healthcare system and urban-suburban setting with well-established community partnerships and logistical infrastructure for produce delivery. These contextual factors, including proximity to partner organizations (e.g., YMCA dietician, regional produce distributors), availability of transportation alternatives, and digital access for virtual coaching, may not be replicable in more rural or under-resourced areas, potentially limiting transferability. Future studies should explore how these findings translate to settings with differing infrastructure, healthcare integration, or population characteristics. Third, while our analysis focused on the participant perspective, we did not include system-level or provider-level viewpoints. These perspectives could offer additional context and enhance understanding of broader implementation dynamics.

### Implications for Future Research

Future research should prioritize both individual and systemic barriers to PRx program effectiveness, particularly for under-resourced communities. While such programs show potential to improve outcomes—such as reductions in blood pressure, improved diabetes management, and improved mental health—sustaining these gains and scaling successful models remain challenges.

First, our findings underscore the value of trusted provider referrals for reaching high-need populations. Future research should explore how PRx screening and referral processes can be systematically embedded in clinical workflows without increasing burden to providers.

Second, future research should identify which combinations of implementation strategies best support sustained engagement. While our findings suggest that pairing practical support with social connection enhanced participation, questions remain about the most effective mix of delivery modes (e.g., in-person, virtual, hybrid) and support mechanisms for different populations. Comparative effectiveness trials could help determine optimal models for diverse community settings.

Third, evaluating sustainable funding models can help with understanding of how funding mechanisms can be adapted and sustained across different state and population contexts [47]. Complementary strategies, including financial subsidies, local food access initiatives, or embedding produce prescriptions into clinical care, may be needed to support long-term behavior change, particularly among economically vulnerable groups.

Finally, more research is needed to understand the long-term impacts of PRx programs on sustained dietary change, improved cardiovascular and mental health outcomes, and overall quality of life [44, 48–50]. Particular attention should be paid to the role of broader social determinants, such as housing, transportation, and social support in shaping program uptake and maintenance [34, 48]. Understanding these contextual factors will be key to refining interventions that can equitably and effectively improve health in high-need populations.

## CONCLUSION

The findings of this study will help inform the design of a future hybrid-effectiveness implementation trial, aimed at evaluating health outcomes and program uptake, engagement, and success. By incorporating the lived experiences of participants, such trials can guide the development of adaptable, community-led, and evidence-based models that address diverse population needs. Advancing interdisciplinary research that combines perspectives from public health, behavioral science, urban planning, community-based organizations, and policy development is essential to help mitigate the structural inequities that limit access to fresh produce. Similarly, exploring innovative delivery models, such as produce prescription programs, can expand reach and accessibility, supporting equitable health outcomes across diverse communities.

## Figures and Tables

**Table 1:** Core Functions of Fresh Food Rx

Core function	Details of core function	Measures used to evaluate outcomes
Produce boxes	Weekly home delivery of produce boxes valued at $25 for 12 months Choice of 3 produce box types each week: (1) mix of produce with eggs, (2) Hispanic inspired produce, (3) mix of produce with no eggs Produce sourced from primarily regional farmers	10-item USDA Food Security Survey Module [53] Dietary Screener Questionnaire [54]Community resource utilization questionnaire
Nutrition counseling by registered dietician	Up to 3 one-on-one counseling sessions tailored to medical needs, current eating patterns and readiness for change First session: in-person at baseline (attendance *n* = 112) Second session: 4–8 weeks after baseline (attendance *n* = 106/112) Third session: 6–8 weeks after second session (attendance *n* = 92/112) Used motivational interviewing techniques to guide participants and set goals for success	Emergency room and hospital admissions and costs for preventable conditions Systolic and diastolic blood pressure, HgbA1C, weight, body mass index, waist circumference Patient Questionnaire-8 [55] Health related-quality of life (HR-QOL-14) [56]
Educational and skill building events	Quarterly community-located (*n* = 3) or virtual (*n* = 1) events designed to increase nutritional knowledge, learn how to purchase healthy foods on a budget, prep and cook seasonal produce, integrate exercise, grow produce Sessions lasted 1–1.5 h Transportation was provided if needed by participants Attendance: Event 1: *n* = 27 Event 2: *n* = 25 Event 3 (virtual): *n* = 2 Event 4: *n* = 19 Of 112 participants completing the study, 46 of 112 participants completed at least 1 event	Satisfaction surveys UCLA Loneliness survey [57]
Monthly newsletters	Distributed inside the produce boxes on last week of each month Nutrition education and tips Recipes using in-season produce	Satisfaction surveys

**Table 2:** Fresh Food Rx Core Functions and Behavioral Strategies Based on BCW and TDF^22,23^

BCW domains	TDF constructs	Barriers	Core function	Behavioral strategy
Capability	Psychological capability Memory, attention, decision processes	Knowledge deficits; lack of education Difficulty in retaining or processing new dietary information	Dietary counseling Education events	3 sessions of dietary counseling focused on assessing current habits, motivational interviewing to set dietary goals, and progress reviews Offer guidance on how to incorporate culturally significant foods into healthier meals Educational events include workshops on meal prep, budgeting, and informed food choices Use visual aids, handouts, and digital reminders to support knowledge retention Break down dietary changes into simple steps and use interactive activities to engage attention and improve decision-making skills
	Physical capability	Cognitive or physical limitations, skill deficits in cooking, lack of experience	Dietary counseling Education events	Assess functional needs related to cooking and meal prep Offer hands-on cooking classes to teach skills like reading food labels and preparing healthy, easy meals Provide recommendations for adaptive tools for those with physical limitations
Opportunity	Physical opportunity	Financial strain; time constraints; physical distance from services (e.g., no nearby grocery)	Produce box delivery Education events	Deliver weekly produce boxes at no cost to participants Collaborate with organizations for financial assistance on food purchases Offer transportation to events, and host online and in person sessions
	Social opportunity	Social isolation Lack of social support	Produce box delivery Education events	Create opportunities for social engagement during education events Offer virtual support groups where participants can share experiences and challenges Referrals to mental health professionals when needed
Motivation	Reflective motivation Belief about capabilities	Negative self-talk; decreased perceived self-efficacy; perceived lack of control	Personalized goal setting with dietician	Implement goal-setting sessions to build self-efficacy Provide positive reinforcement for achieved goals Offer ongoing education on the benefits of healthy eating to empower participants
	Optimism Reinforcement	Lack of well-defined health goals or intrinsic motivation to pursue dietary changes	Personalized health plans	Develop personalized, measurable dietary goals with regular check-ins Focus on motivation improvement and provide strategies for self-care Use behavioral nudges such as reminder calls or texts to encourage continued use of the produce box and adherence to dietary goals Positive reinforcement after goal achievements via small incentives

**Table 3:** Interview Guide Based on RE-AIM

RE-AIM domains	Question
Essence of experience	Prior to enrollment in this program, can you please describe the ways that you went about buying healthy foods? What, if anything, made it difficult to buy healthy foods?
Effectiveness	How do you think having access to fresh produce has impacted your health? Can you tell me an example of how your eating habits have changed since enrolling in the program? Can you describe any health-related changes that you have noticed since engaging in this program?
Reach/adoption	What made you interested in enrolling the Fresh Food Rx program? Prompt: What did you think the Fresh Foods program could help you with? What do you believe are the best ways for our team to tell people about this program or reach additional participants in the future? Prompt: What has motivated you to continue participating in the program?
Implementation	How many nutrition coaching sessions did you participate in? If no, why didn’t you participate? What prevented you from participating? How do you feel about the health/nutrition coaching sessions that are a part of this program? Prompts: How do you feel about the length of the sessions? How do you feel about the location of the sessions? Prompt: What are some aha moments that you have had (or things you have learned) about healthy eating or self-care from these sessions? What ways can we improve the health coaching program? Did you attend one or both events? If no, what prevented you from attending? How did you feel about the healthy eating events that are a part of the Fresh Food Rx program? Prompt: What are some things you have learned about health, wellbeing, or healthy eating from these events? What other ways can a program like FFRx support you with your health or wellbeing?
Maintenance	What is the likelihood that you will continue to participate in the produce delivery program after the study is over? Why or why not? If not, what are the barriers to you continuing to receive produce delivery after the study is finished? How do you feel about the cost of participating in the produce delivery program after the study? How likely are you to continue eating fresh produce on a regular basis after the study is over? Why or why not?
Closing reflection	What are other things that you feel are important for us to know?

**Table 4:** Demographics

	Participant *n* (%)
Sex	
* Female*	10 (67%)
* Male*	5 (23%)
Age	
* 45–54*	2 (13%)
* 55–64*	10 (67%)
* 65–74*	3 (20%)
Race/ethnicity	
* Black*	7 (47%)
* White*	6 (40%)
* Hispanic*	2 (13%)

**Table 5:** Core Functions Mapped to Themes and Subthemes

RE-AIM^24^ domain	Core function	Theme	Subtheme	Impactful quotes
Reach		Participants expressed a variety of motivations for participation	Financial relief and resource allocation Participants’ motivation to improve health Referrals from trusted sources	“I figured if it was voluntary, and there was some benefit in terms of getting fresh produce, why not?” (P10) I was interested in seeing how it would help me with my overall health.” (P11) “I heard it from my doctor…she said it was a good program.” (P09)
Effectiveness	Coaching, educational events	Improved dietary knowledge, community engagement, observed improvements in health, and motivations to prioritize self-care were key benefits of program participation	Paricipants gained improved dietary knowledge and skills during program participation	“I learned that the way you cook your food makes a difference…I like to do new recipes. I like to see how other people do stuff and adapt what they do to see if it will help me.” (P05) “The education classes and work-shops… increase your knowledge about how to combine fresh things with other foods and make it a well-balanced and nutritious meal.” (P04)
	Coaching, educational events		Participants were motivated to prioritize self-care and expressed improvements in physical and mental health Participants experienced an improved sense of community and social connections	“Because of losing some of the weight, the knee pain, the joint pain, I don’t have that like I used to have. I just feel more energetic.” (P04) “I like connecting with other people. I might meet a buddy who wants to exercise with me…I want to make friends who the same frame of mind” (P01)
	Produce box		Program benefits extended to others	My daughter, she lives with me, and she was real encouraged on trying new things for us to eat instead of having the same old thing all the time and having abundance. (P11)
Adoption	Coaching, educational event	Several factors influenced program engagement including personal connections, financial benefits, and sustained access to weekly produce	Personal connections made through nutrition coaching and educational events deepened engagement with the program	“She [dietician] was very positive. It was very motivational… [the education event] was fun and meetin’ the other people that was in it was great.” (P03)
	Produce boxes		The weekly delivery of fresh produce boxes provided participants with stability and incentive to maintain healthy eating habits	“It helps me be more aware of what I’m eating, what I’m putting in my body. Like I said, it’s a blessing for me. It encouraged me to try new recipes, to do things that I wouldn’t even do.” (P05) The thing I like about this program is that it offers food from different parts of this state and whether the climate as diverse as this you can get all different kinds of things during the year. (P10)
	Produce boxes		The program’s financial benefits alleviated economic burden	“That box is helping me a whole lot. That’s the mental part, not worrying about having and not having.” (P16)
Implementation		While participants recognized the program’s benefits, several barriers hindered full participation		“Telephone was a lot better for me because of the financial. I live on social security and the gas it takes to get there.” (P08)
Maintenance		Participants expressed a strong commitment to maintaining healthy eating habits after the program’s discontinuation, but many were unsure how they would do so due to systemic barriers to healthy food access		I’ll be eating just the way I am now, everything just healthy. Im’ma continue eating fruits and vegetables like I do now. I got used to eating it. I’m used to it now, so I have to do it. (P19)

## Data Availability

Data is available upon request.
